# TIVAN-indel: a computational framework for annotating and predicting non-coding regulatory small insertions and deletions

**DOI:** 10.1093/bioinformatics/btad060

**Published:** 2023-01-27

**Authors:** Aman Agarwal, Fengdi Zhao, Yuchao Jiang, Li Chen

**Affiliations:** Department of Computer Science, Indiana University, Bloomington, IN 47405, USA; Department of Biostatistics, University of Florida, Gainesville, FL 32603, USA; Department of Biostatistics, University of North Carolina, Chapel Hill, NC 27516, USA; Department of Biostatistics, University of Florida, Gainesville, FL 32603, USA

## Abstract

**Motivation:**

Small insertion and deletion (sindel) of human genome has an important implication for human disease. One important mechanism for non-coding sindel (nc-sindel) to have an impact on human diseases and phenotypes is through the regulation of gene expression. Nevertheless, current sequencing experiments may lack statistical power and resolution to pinpoint the functional sindel due to lower minor allele frequency or small effect size. As an alternative strategy, a supervised machine learning method can identify the otherwise masked functional sindels by predicting their regulatory potential directly. However, computational methods for annotating and predicting the regulatory sindels, especially in the non-coding regions, are underdeveloped.

**Results:**

By leveraging labeled nc-sindels identified by cis-expression quantitative trait loci analyses across 44 tissues in Genotype-Tissue Expression (GTEx), and a compilation of both generic functional annotations and large-scale epigenomic profiles, we develop TIssue-specific Variant Annotation for Non-coding indel (TIVAN-indel), which is a supervised computational framework for predicting non-coding regulatory sindels. As a result, we demonstrate that TIVAN-indel achieves the best prediction performance in both with-tissue prediction and cross-tissue prediction. As an independent evaluation, we train TIVAN-indel from the ‘Whole Blood’ tissue in GTEx and test the model using 15 immune cell types from an independent study named Database of Immune Cell Expression. Lastly, we perform an enrichment analysis for both true and predicted sindels in key regulatory regions such as chromatin interactions, open chromatin regions and histone modification sites, and find biologically meaningful enrichment patterns.

**Availability and implementation:**

https://github.com/lichen-lab/TIVAN-indel

**Supplementary information:**

Supplementary data are available at *Bioinformatics* online.

## 1 Introduction

Small insertion and deletion (sindels) (e.g. <50 bp) are the second most common mutations in the human genome, which make up 15–21% of human polymorphism (Sayers *et al.*, 2019), only after single-nucleotide variants (SNVs). In the coding regions, sindels can alter the protein sequence by adding/removing multiples of three nucleotides, which leads to the insertion/deletion of one or more amino acids (non-frameshift sindels) or adding/removing a number of base pairs that is not a multiple of 3, which consequently disrupts the triplet reading frame of a DNA sequence (frameshift sindels). Recent studies have shown that coding sindels can significantly influence a range of phenotypic and molecular effects (Garcia-Diaz and Kunkel, 2006; Montgomery *et al.*, 2013). For example, cystic fibrosis, one common human disease, is frequently caused by a coding sindel within the CFTR gene that eliminates a single amino acid (Collins *et al.*, 1987). Moreover, non-coding sindels (nc-sindels) have also been found to play an important role in human traits and diseases by affecting the gene expression such as altering the pattern, phasing and spacing of DNA sequences within promoters (Cheung and Spielman, 2009; Lee *et al.*, 2018). One example is that DNA insertions within the promoter region of the FMR1 gene cause Fragile X syndrome in humans (Warren *et al.*, 1987). However, the functional consequence of nc-sindels has been less explored compared with their coding counterparts.

Despite the experimental validation and clinical interpretation of sindels in the past decades, the knowledge of sindels is mostly limited to thousands of pathogenic sindels in Human Gene Mutation Database (HGMD) (Cooper *et al.*, 1998) and ClinVar (Landrum *et al.*, 2016). However, the functional consequences of the majority of discovered indels such as those in gnomAD v2.1 (Karczewski *et al.*, 2020) and FAVOR (Zhou *et al.*, 2022) are still unknown. Though *in silico* prediction provides an alternative way to understand the functional consequence of non-coding genetic variations, most computational methods focus on predicting the functional consequence of non-coding SNVs (Chen *et al.*, 2016, 2019, 2022; Chen and Qin, 2017; Ionita-Laza *et al.*, 2016; Li *et al.*, 2022a; Ritchie *et al.*, 2014; Vitsios *et al.*, 2021; Wang and Chen, 2022; Wang *et al.*, 2021). Nevertheless, only a few methods have been developed for predicting functional nc-sindels. To name a few, CADD adopts support vector machine (SVM) to integrate diverse genomic annotations such as sequence context, evolutionary constraints and epigenetic measurements for observed and simulated sindels (Kircher *et al.*, 2014) into a single score, which ranks the deleteriousness of sindels in both coding and non-coding regions. FATHMM-indel, another SVM-based machine learning approach, adopts a similar training feature set as CADD, to exclusively predict pathogenic sindels in the non-coding regions (Ferlaino *et al.*, 2017) by using the pathogenic nc-sindels from HGMD as the training samples. Recently, deep learning methods have been developed to predict functional non-coding variants, which include both SNVs and sindels. Representative work such as DeepSEA (Zhou and Troyanskaya, 2015) and DanQ (Quang and Xie, 2016) use the genomic sequence to simultaneously predict thousands of chromatin-profiling data, which include transcription factor binding, histone-mark profile and open chromatin across multiple cell types. Using the predicted chromatin-profiling data from both reference and alternative genomic sequences, DeepSEA adopts a boosted logistic regression classifier to discriminate non-coding pathogenic sindels in HGMD and background sindels from the 1000 Genomes Project (Auton *et al.*, 2015).

Regulation of gene expression is a key molecular mechanism for nc-sindels to achieve phenotypic and clinical significance by disrupting the normal sequence pattern in the promoters and enhancers, which can influence the nearby gene expression (Abraham *et al.*, 2017; Cheung and Spielman, 2009; Warren *et al.*, 1987). Similar to SNVs, the same challenge is faced by current sequencing experiments to identify nc-indels that regulate gene expression due to the small sample size, low minor allele frequencies (MAFs) and small effect size. Moreover, existing computational methods, such as CADD, FATHMM-indel and DeepSEA, focus on predicting the pathogenicity or deleteriousness of nc-sindels, rather than the molecular impact. Therefore, it is crucial to develop a computational tool for predicting the functional potential of nc-sindels that may alter gene expression, which can provide a mechanistic understanding of the functional role of nc-indels in disease pathogenesis.

In this work, we develop a novel computational framework, named TIssue-specific Variant Annotation for Non-coding indel (TIVAN-indel), which aims to predict nc-sindels that potentially regulate gene expression. Our contribution mainly lies in two aspects. First, to our best knowledge, TIVAN-indel is the first computational tool to predict tissue-specific regulatory nc-sindels. Second, TIVAN-indel leverages both generic functional annotations (e.g. evolutionary score and sequence characteristics) and large-scale epigenomic profiles in a supervised machine learning framework to improve the prediction performance. We benchmark TIVAN-indel and existing approaches on 44 tissues in Genotype-Tissue Expression (GTEx) using a cross-validation within-tissue approach and an independent cross-tissue approach, respectively. As an independent evaluation, we train TIVAN-indel from the ‘Whole Blood’ tissue in GTEx and test the model using 15 immune cell types from an independent study Database of Immune Cell Expression (DICE). To demonstrate nc-sindels play a key role in gene regulation, we perform an enrichment analysis for both labeled and predicted nc-sindels in key regulatory regions such as chromatin interactions, open chromatin regions and histone modification sites, and find biologically meaningful enrichment patterns.

## 2 Materials and methods

### 2.1 Creating the training samples

Using a similar approach as our previously developed method TIVAN (Chen *et al.*, 2019), we obtain tissue-specific nc-sindels, which are derived from the cis-expression quantitative trait loci (eQTLs) analysis in GTEx (Aguet *et al.*, 2020). For each tissue, we create the positive set by selecting the nc-sindels from GTEx (v6p), which meet the following criteria: (i) nc-sindels less than 100 bp; (ii) distance to transcription start site (TSS) less than 100 kb as a nc-sindel may influence nearby gene expression and (iii) *q*-value less than 0.05 with at least one associated gene. To create the negative set, we select the nc-sindels from GTEx (v7), which meet the following criteria: (i) non-overlap with any nc-sindel in the positive set; (ii) nc-sindels less than 100 bp; (iii) associated with at least one gene in the positive set; (iv) distance to the TSS less than 100 kb; (v) *q*-value larger than 0.2 and (vi) with a matched empirical distribution of MAF as the nc-sindels in the positive set. Without loss of generality, we create a balanced negative set. The sample sizes of the positive sets across 44 tissues are summarized in Supplementary Table S1.

### 2.2 Creating the functional annotations

We create the functional annotations from two sources. The first source is the functional annotations curated in the latest CADD v1.6 (Genome build GRCh37/hg19) (Rentzsch *et al.*, 2019). We only keep functional annotations with less than 10% missing values and impute the missing values using the median of non-missing values for each annotation. As a result, we have 45 functional annotations, which mainly include (i) base-level features such as conservation score and GC content; (ii) chromatin states in 48 cell types; (iii) H3K27ac, H3K4me1 and H3K4me3 levels and (iv) number of frequent, rare and single occurrence TOPMed SNVs in BRAVO server (Taliun *et al.*, 2021) at 100, 1000 and 10 000 bp resolution. The detailed descriptions of the 45 functional annotations can be found in Supplementary Table S2.

The second source is the collection of large-scale epigenomic profiles, which are predicted from a sequence-based deep learning model. First, we obtain a fixed length of 1000 bp reference genomic sequence of each sindel. Similarly, we create the alternative genomic sequence by changing the reference allele to the alternative allele. Each genomic sequence is one-hot encoded as ‘A’—[1, 0, 0, 0], ‘G’—[0, 1, 0, 0], ‘C’—[0, 0, 1, 0] and ‘T’—[0, 0, 0, 1]. Next, we exploit a recently developed multi-task convolutional neural network named DanQ (Quang and Xie, 2016), which takes the one-hot encoded reference/alternative genomic sequence as the input to simultaneously predict large-scale chromatin-profiling data, including transcription factor binding, histone mark profile and open chromatin across multiple cell types. As a result, DanQ predicts 919 cell type-specific binary epigenomic profiles, which include TF binding site (690), DNase I hypersensitive site (125) and histone modification sites (104) (Quang and Xie, 2016). A total of 919 + 919 = 1838 predicted epigenomic profiles are used as the functional annotations for both reference and alternative alleles of each sindel. It should be noted that other deep learning models such as DeepSEA (Zhou and Troyanskaya, 2015) can also be used for the same purpose. We adopt DanQ in this work as it has been demonstrated to outperform DeepSEA in the prediction task (Quang and Xie, 2016). Collectively, both 45 generic functional annotations derived from CADD and 1838 epigenomic profiles predicted by DanQ will be used to annotate the labeled nc-sindels as the training features.

### 2.3 Computational framework of TIVAN-indel

**Fig. 1.**
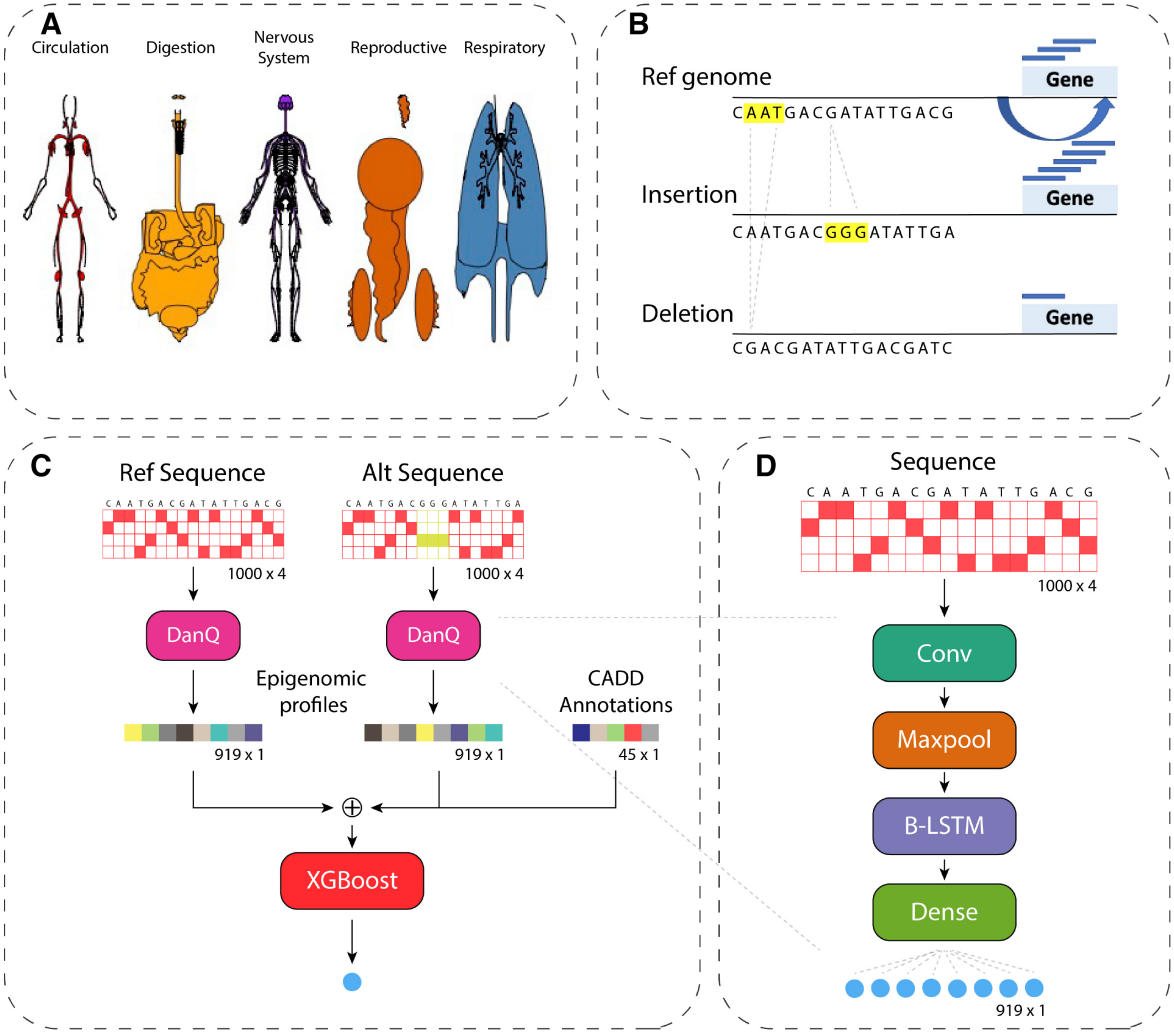
Overview of TIVAN-indel. (**A**) We create the positive and negative nc-sindels, which are derived from the cis-eQTL analysis, across 44 tissues in GTEx. (**B**) A cartoon example to demonstrate that a small insertion/deletion in the non-coding region can affect nearby gene expression. (**C**) TIVAN-indel takes both generic functional annotations derived from CADD and epigenomic profiles predicted by DanQ from both reference and alternative genomic sequences to annotate the labeled nc-sindels as the training features. Finally, TIVAN-indel adopts XGBoost for the binary classification. (**D**) A brief illustration of the model architecture of the sequence-based deep learning model (e.g. DanQ)

As shown in Figure 1, the computational framework of TIVAN-indel mainly consists of two steps. The first step is feature extraction. Given the genomic loci of a nc-sindel, TIVAN-indel will obtain 45 functional annotations derived from CADD and 919 epigenomic profiles predicted by DanQ from both reference and alternative genomic sequence, respectively. The second step is model training. TIVAN-indel will adopt XGBoost as the supervised approach to train the prediction model. It should be noted that TIVAN-indel is trained in a tissue-specific way and each tissue has its own TIVAN-indel model.

### 2.4 Promoter-centered captured HiC data

To calculate the enrichment of nc-sindels in chromatin interactions, we collect a deposition of promoter-centered chromatin interactions in 3D-genome Interaction Viewer and database (3DIV) (Yang *et al.*, 2018), which has profiled a comprehensive set of promoter-centered capture Hi-C datasets (pcHi-C) across 27 human tissues in 4 categories including embryonic stem cell, 4 early embryonic lineages, 2 primary cell lines and 20 primary tissue types. For each tissue, pcHi-C provides both promoter–enhancer (PE) interactions and promoter–promoter (PP) interactions. Next, we adopt a similar procedure to create positive and negative chromatin interactions as in our previous work (Agarwal and Chen, 2023). Specifically, we filter chromatin interactions with distance more than 10^6^ and consider only intra-chromosomal interactions. We further format each anchor into 2 kb region with 1 kb upstream and downstream of the midpoint of the anchor. Among all interactions, we label significant interactions (FDR <0.1) as the positive set and only keep tissue with more than 100 significant interactions. As a result, the number of positive samples ranges from 972 to 56 057 with a median of 5071 among 26 tissues for PE and from 187 to 10 338 with a median of 917 among 25 tissues for PP. To create the negative set, we select interactions (FDR >0.5) with matched GC contents as the positive set. Without loss of generality, the number of negative interactions is set as the same as the positive interactions. More details of common tissues between GTEx and pcHi-C in 3DIV can be found in Supplementary Table S3.

### 2.5 Roadmap epigenomics data

To calculate the enrichment of nc-sindels in the epigenomic regions, we collect open chromatin and histone mark ChIP-seq data from Roadmap Epigenomics (Kundaje *et al.*, 2015) in common tissues between GTEx and Roadmap Epigenomics (Supplementary Table S4). For active histone marks, we collect H3K4me3 and H3K9ac associated with promoter activation, H3K4me1 and H3K27ac associated with enhancer activation, H3K36me3 associated with active gene body (Chantalat *et al.*, 2011) and H3K27me3 and H3K9me3 associated with gene repression. To create the positive set, we keep statistically significant ChIP-seq peak regions (FDR <0.05) as the positive set and fix the peak size into 1000 bp by extending the peak center upstream and downstream 500 bp. To create the negative set, we randomly choose the same number of 1000 bp windows across the whole genome with a matched GC content distribution as the positive set.

## 3 Results

### 3.1 Integrating both generic functional annotations and large-scale epigenomic profiles improves the prediction performance for the regulatory non-coding small indels

Conventional computational methods for predicting regulatory nc-sindels such as CADD and FATHMM-indel only adopt tens of genomic annotations as the training features. By assuming that regulatory nc-sindels can perform a biological function in the epigenomic regions, we hypothesize that including these epigenomic data as additional training features can improve the prediction performance for the regulatory nc-sindels.

By leveraging DanQ, which is a deep learning approach that can predict hundreds of epigenomic profiles simultaneously given the genomic sequence as the input, we obtain 919 predicted cell type-specific epigenomic profiles for each nc-sindel. Since a functional nc-sindel is assumed to disrupt the normal sequence pattern, which may cause the change of epigenomic profile and indicate the functional consequence of the nc-sindel, we modify each reference sequence into an alternative sequence by shifting the sequence downstream for the insertion and upstream for the deletion to make the alternative genomic sequence has the same length as the reference one. Accordingly, we also obtain 919 predicted cell type-specific epigenomic profiles from the alternative genomic sequence.

To demonstrate the necessity to include both generic functional annotations derived from CADD and epigenomic profiles predicted by DanQ from both reference and alternative genomic sequence, we use 5-fold cross-validation on 44 tissues in GTEx to evaluate three feature sets of TIVAN-indel: (i) 45 CADD functional annotations; (ii) 45 CADD functional annotations + 919 multi-omics features under reference sequence and (iii) 45 CADD functional annotations + 919 multi-omics features under both reference and alternative sequence. We report the AUROC and AUPRC for each tissue and use the median of AUROC and AUPRC across 44 tissues to compare the prediction performance among three feature sets.

**Fig. 2.**
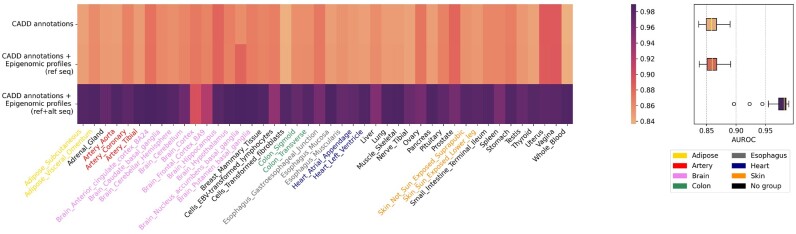
Comparison of three feature sets of TIVAN-indel: (i) 45 CADD annotations; (ii) 45 CADD annotations + 919 epigenomic profiles predicted from the reference genomic sequence and (iii) 45 CADD annotations + 919 × 2 epigenomic profiles predicted from both the reference and alternative genomic sequence. We evaluate the prediction performance using 5-fold cross-validation across 44 tissues in GTEx. The median of AUROC across 44 tissues is reported for each feature set

As a result (Fig. 2 and Supplementary Fig. S1), the inclusion of epigenomic features predicted from the reference genomic sequence will slightly improve both the median of AUROC [CADD annotations: 0.858, CADD annotations + epigenomic profiles (ref seq): 0.859] and the median of AUPRC [CADD annotations: 0.843, CADD annotations + epigenomic features (ref seq): 0.845]. This observation indicates that the inclusion of epigenomic profiles will improve the prediction performance as a regulatory nc-sindel can be biologically functional in the epigenomic regions. Importantly, further including epigenomic profiles predicted from the alternative genomic sequence [CADD annotations+epigenomic features (ref seq+alt seq)], the prediction performance is significantly improved by achieving the highest median of AUROC (0.982) and AUPRC (0.984). Indeed, it is the comparison of the predicted epigenomic profiles between the reference and alternative genomic sequence that characterizes the functional consequence of a regulatory nc-sindel. In the following sections, we will use all three feature sets as the default setting for TIVAN-indel.

### 3.2 Evaluating TIVAN-indel for predicting non-coding regulatory small indels using a within-tissue approach

We evaluate TIVAN-indel and the competing methods using labeled nc-sindels from 44 tissues in GTEx. For TIVAN-indel, we adopt 5-fold cross-validation to obtain the prediction score for each fold of the 5-folds for each tissue. The prediction scores of the 5-folds are concatenated and compared with the true labels. For CADD and FATHMM-indel, we obtain the genome-wide prediction scores for labeled nc-sindels from CADD web server (https://cadd.gs.washington.edu/score) and FATHMM-indel web server (http://indels.biocompute.org.uk/). The precomputed scores are compared with the true labels directly. As a result, we find TIVAN-indel performs much better than CADD and FATHMM-indel for each tissue (Supplementary Figs S2 and S3). However, the above comparison may be biased. Though the testing sets from the three methods are all from labeled nc-sindels in GTEx, the training sets are different. TIVAN-indel uses labeled nc-sindels from the same tissue in GTEx, CADD adopts high-frequency human-derived alleles and FATHMM-indel utilizes pathogenic nc-sindels from HGMD. The difference of the training sets makes CADD and FATHMM-indel favorable on predicting the pathogenicity or deleteriousness of nc-sindels rather than molecular impact on gene expression.

To have a fair comparison, we train and test CADD and FATHMM-indel in the same way as TIVAN-indel using 5-fold cross-validation. Since both CADD and FATHMM-indel adopt SVM and share a similar feature set, we use SVM to train a classifier and use CADD annotations as the training features. We denote the trained SVM as ‘CADD/FATHMM-indel (SVM)’, which serves as the representative for both CADD and FATHMM-indel. In addition, we implement a common approach of deep learning models to predict functional non-coding variants. Specifically, we take the absolute and relative difference of predicted epigenomic profiles from reference and alternative genomic sequence as the training features in a boosted logistic regression model (Zhou and Troyanskaya, 2015). We denote this approach as ‘DanQ/DeepSEA (Boosted LR)’.

**Fig. 3.**
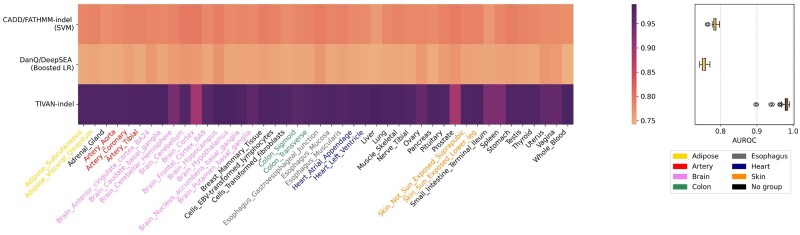
Comparison between TIVAN-indel, CADD/FATHMM-indel (SVM) and DanQ/DeepSEA (Boosted LR) using the within-tissue approach for 44 tissues in GTEx. Each method is trained and tested using 5-fold cross-validation for each tissue. AUROC is reported for each tissue

Consequently, TIVAN-indel still outperforms CADD/FATHMM-indel (SVM) and DanQ/DeepSEA (Boosted LR) (Fig. 3 and Supplementary Fig. S4) by achieving the highest median of AUROC and AUPRC (AUROC: 0.983 versus 0.784 versus 0.753; AUPRC: 0.986 versus 0.840 versus 0.816). The significant performance improvement of TIVAN-indel may be attributed to two reasons. First, XGBoost, adopted by TIVAN-indel, has been demonstrated to be more powerful than conventional logistic regression (Dwidarma *et al.*, 2021) and SVM (Husna *et al.*, 2020). Second, TIVAN-indel leverages the strength of both CADD annotations and large-scale epigenomic profiles. In contrast, CADD/FATHMM-indel (SVM) only considers CADD annotations, while DanQ/DeepSEA (Boosted LR) mainly focuses on epigenomic profiles.

### 3.3 Evaluating TIVAN-indel for predicting non-coding regulatory small indels using a cross-tissue approach

We further perform a cross-tissue approach, where all methods are trained using labeled sc-indels from one tissue and tested using labeled sc-indels from another tissue. As the training set and testing set are from different tissues, the cross-tissue approach provides a more objective evaluation for all methods. Accordingly, we train TIVAN-indel, CADD/FATHMM-indel (SVM) and DanQ/DeepSEA (Boosted LR) using one tissue and test the model on the other 43 tissues. The overlapped labeled nc-sindels between the training and the testing sets are removed from the testing set.

**Fig. 4.**
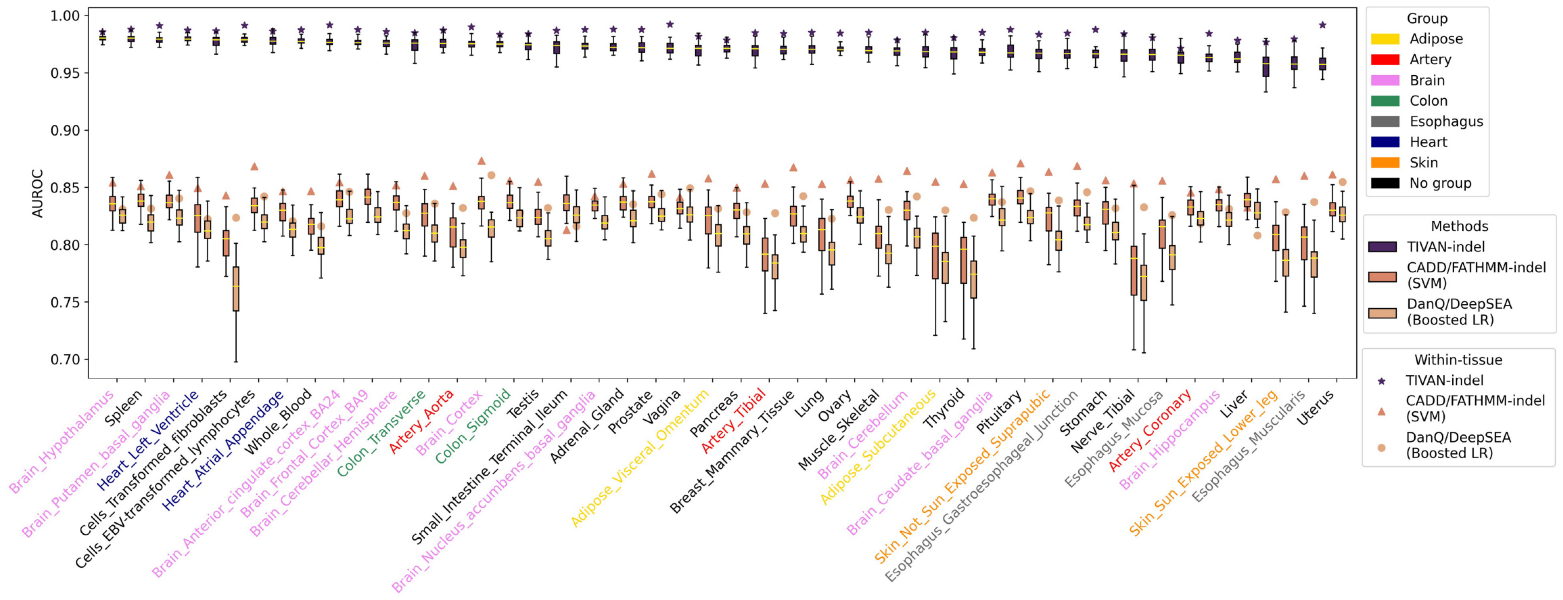
Comparison between TIVAN-indel, CADD/FATHMM-indel (SVM) and DanQ/DeepSEA (Boosted LR) using the cross-tissue approach for 44 tissues in GTEx, where each method is trained using one tissue and tested on the other 43 tissues. The overlapped nc-sindels between the training and testing sets are removed from the testing set. For each tissue, AUROC is reported for the other 43 tissues as demonstrated in the boxplot. The asterisk denotes the AUROC calculated from the within-tissue approach. In total, 44 tissues are colored in 8 tissue groups

In the cross-tissue evaluation, we order 44 tissues based on the median of AUROC and AUPRC of TIVAN-indel (Fig. 4 and Supplementary Fig. S5) and have three important findings. First, TIVAN-indel still outperforms CADD/FATHMM-indel (SVM) and DanQ/DeepSEA (Boosted LR) for all tissues and achieves the highest median of AUROC and AUPRC (AUROC: 0.971 versus 0.831 versus 0.813; AUPRC: 0.964 versus 0.774 versus 0.722), which demonstrates the superiority of TIVAN-indel in the cross-tissue evaluation. Second, for all methods, within-tissue prediction achieves overall higher AUROC and AUPRC than cross-tissue prediction due to the tissue-matching between training and testing sets. Moreover, the advantage of within-tissue prediction compared with cross-tissue prediction is more evident for CADD/FATHMM-indel (SVM) and DanQ/DeepSEA (Boosted LR). Third, we find top-performed tissues for TIVAN-indel mainly come from the Brain category.

### 3.4 Evaluating TIVAN-indel for predicting non-coding regulatory small indels in an independent study

We have demonstrated that TIVAN-indel outperforms competing methods using both within-tissue and cross-tissue approaches on the GTEx data. We further perform an independent evaluation, where the model is trained on the GTEx data and tested on another QTL study named Database of Immune Cell Expression (DICE) (eQTLs and Epigenomics) (https://dice-database.org/) (Schmiedel *et al.*, 2018). To create the testing set, we collect cell-type specific regulatory nc-sindels from 15 immune cell types in DICE, which include classic and non-classic monocytes, NK cells, naive B cells, naive/stimulated CD4+ T cells, naive/stimulated CD8+ T cells, naive/memory Treg cells, Th1 cells, Th17 cells, Th1/17 cells, Th2 cells and Tfh. Using the same data processing steps for the GTEx data, we create the nc-sindels in the positive and negative sets for each immune cell type. The numbers of regulatory nc-sindels for the 15 immune cell types are summarized in Supplementary Table S5. Because immune cell types can be found in peripheral blood, we train the model from the ‘Whole blood’ in GTEx and test the model on the 15 immune cell types in DICE.

**Fig. 5.**
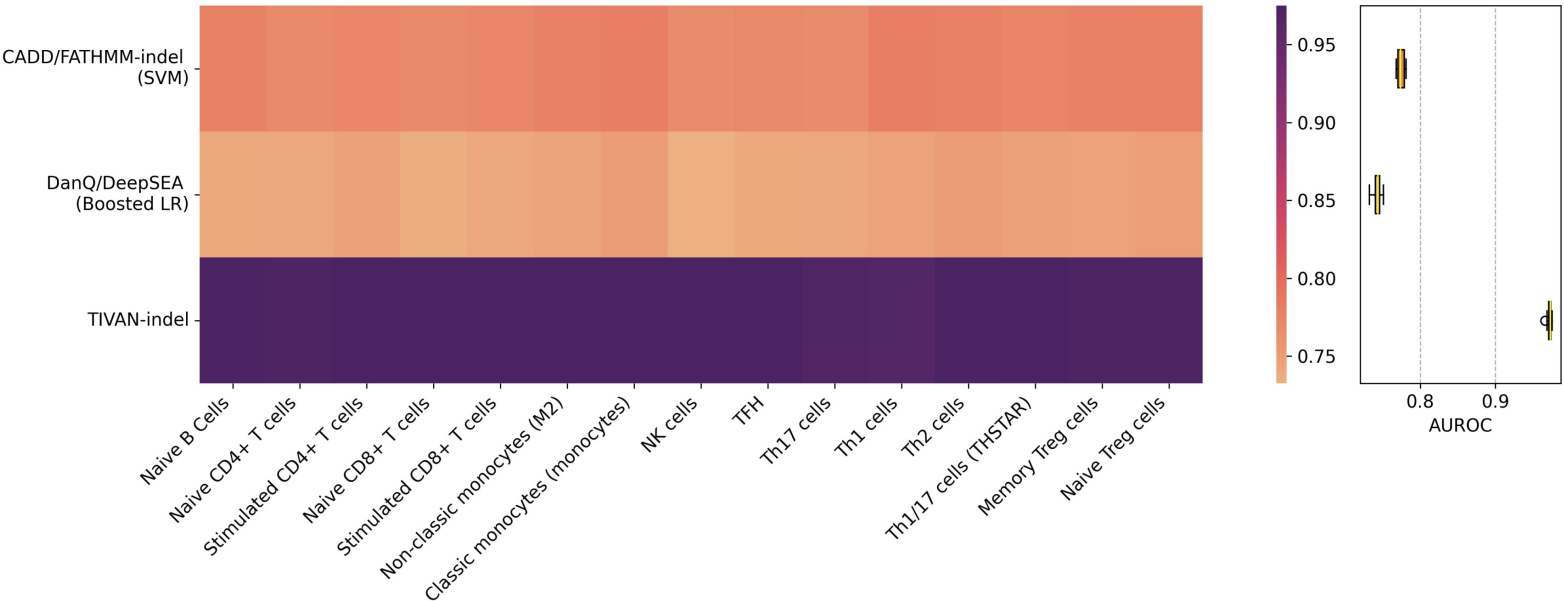
Comparison between TIVAN-indel, CADD/FATHMM-indel (SVM) and DanQ/DeepSEA (Boosted LR) by training the model on the ‘Whole blood’ in GTEx and testing the model on 15 immune cell types in DICE. The overlapped nc-sindels between training and testing sets are removed from the testing set. The AUROC is reported for each cell type

Similarly, TIVAN-indel consistently performs better than CADD/FATHMM-indel (SVM) and DanQ/DeepSEA (Boosted LR) for all 15 immune cell types and achieves the highest median of AUROC (0.973 versus 0.773 versus 0.744) and AUPRC (0.978 versus 0.792 versus 0.733) (Fig. 5 and Supplementary Fig. S6). The accurate prediction of all methods indicates the biological connection between whole blood and immune cell types. Moreover, the significant improvement of prediction performance of TIVAN-indel in the independent evaluation further strengthens the superiority of using TIVAN-indel to predict the regulatory nc-sindels.

### 3.5 Evaluating TIVAN-indel for predicting pathogenic non-coding small indels

Though TIVAN-indel is developed to predict regulatory nc-indels, we hereby evaluate whether TIVAN-indel can be extended to predict pathogenic nc-sindels, which is the main task for CADD and FATHMM-indel. To do this, we download the latest version of ‘variant_summary.txt.gz’ file from clinvar ftp website (https://ftp.ncbi.nlm.nih.gov/pub/clinvar/tab_delimited/). We collect small indels less than 100 bp in the non-coding regions (hg19/GRCh37), which are annotated with ‘Insertion’, ‘Deletion’, ‘Indel’ or ‘Duplication’. We define the pathogenic nc-indels with ‘ClinicalSignificance’ noted as ‘Pathogenic’ and the benign nc-sindels with ‘ClinicalSignificance’ noted as ‘Benign’. In addition, we only select highly confident nc-sindels with the ‘Review status’ noted as ‘multiple submitters, no conflicts’ or ‘reviewed by expert panel’. Finally, we have 132 pathogenic nc-sindels and 1104 benign nc-sindels.

**Fig. 6.**
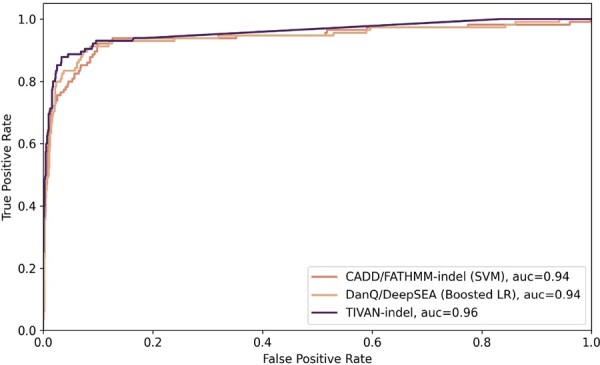
Comparison between TIVAN-indel, CADD/FATHMM-indel (SVM) and DanQ/DeepSEA (Boosted LR) in predicting pathogenic nc-sindels in ClinVar. The AUROC is reported

Similar to other work (Ferlaino *et al.*, 2017), we also adopt 5-fold cross-validation to evaluate all methods. As a result, TIVAN-indel still outperforms CADD/FATHMM-indel (SVM) and DanQ/DeepSEA (Boosted LR) by achieving the highest AUROC (Fig. 6) and AUPRC (Supplementary Fig. S7). The observation indicates the robustness of TIVAN-indel, which can accurately predict both regulatory nc-sindels that have an impact on the nearby gene expression and pathogenic nc-sindels that have a clinical impact.

### 3.6 Enrichment analysis of regulatory nc-sindels in key regulatory regions

We hypothesize that regulatory nc-sindels need to be enriched in key regulatory regions such as enhancer, promoter and chromatin interaction in order to have an impact on gene expression. Moreover, we assume that a good prediction of regulatory nc-sindels should achieve a similar or better enrichment than that of true nc-sindels in the regulatory regions. Thus, we perform the enrichment analysis for both true and predicted nc-sindels.

To obtain key regulatory regions, we collect tissue-specific pcHi-C data across 27 human tissues from 3DIV (Yang *et al.*, 2018) and ChIP-seq data profiling open chromatin and key histone marks from Roadmap Epigenomics (Kundaje *et al.*, 2015). For pcHi-C data, we create tissue-specific positive/negative PE interactions and PP interactions as our previous work (Agarwal and Chen, 2023). For ChIP-seq data, we treat peak regions with the FDR less than 0.05 as the positive set and randomly sampled regions with a matched GC content distribution as the negative set. More details of the data collection and processing can be found in Section 2. We perform the enrichment analysis in a tissue-specific way by matching the tissues in GTEx, pcHi-C data and ChIP-seq data.

In the enrichment analysis, we calculate the number of positive/negative nc-sindels in the positive/negative regulatory regions. For peak regions, we directly overlap the genomic locations of nc-sindels and genomic locations of the peak regions. For chromatin interactions, we overlap the genomic locations of nc-sindels and the genomic locations of the two anchors of each chromatin interaction. As long as a nc-sindel hits one of the two anchors, we consider the nc-sindel is in the chromatin interaction. As a result, we obtain *n*_11_: number of the positive nc-sindels in the positive regulatory regions; *n*_12_: number of the positive nc-sindels in the negative regulatory regions; *n*_21_: number of the negative nc-sindels in the positive regulatory regions; and *n*_22_: number of the negative nc-sindels in the negative regulatory regions. Then, we perform a Fisher’s exact test to calculate the enrichment and report *P*-values by assuming that the positive nc-sindels are more enriched in the positive regulatory regions. The enrichment is defined as the odds ratio in the formula as $\text{OR}=\frac{\frac{n_{11}}{n_{12}}}{\frac{n_{21}}{n_{22}}}$. Similarly, we calculate the enrichment using predicted labels. By default, the predicted labels are defined by a threshold of 0.5 to classify positive/negative nc-sindels.

**Fig. 7.**
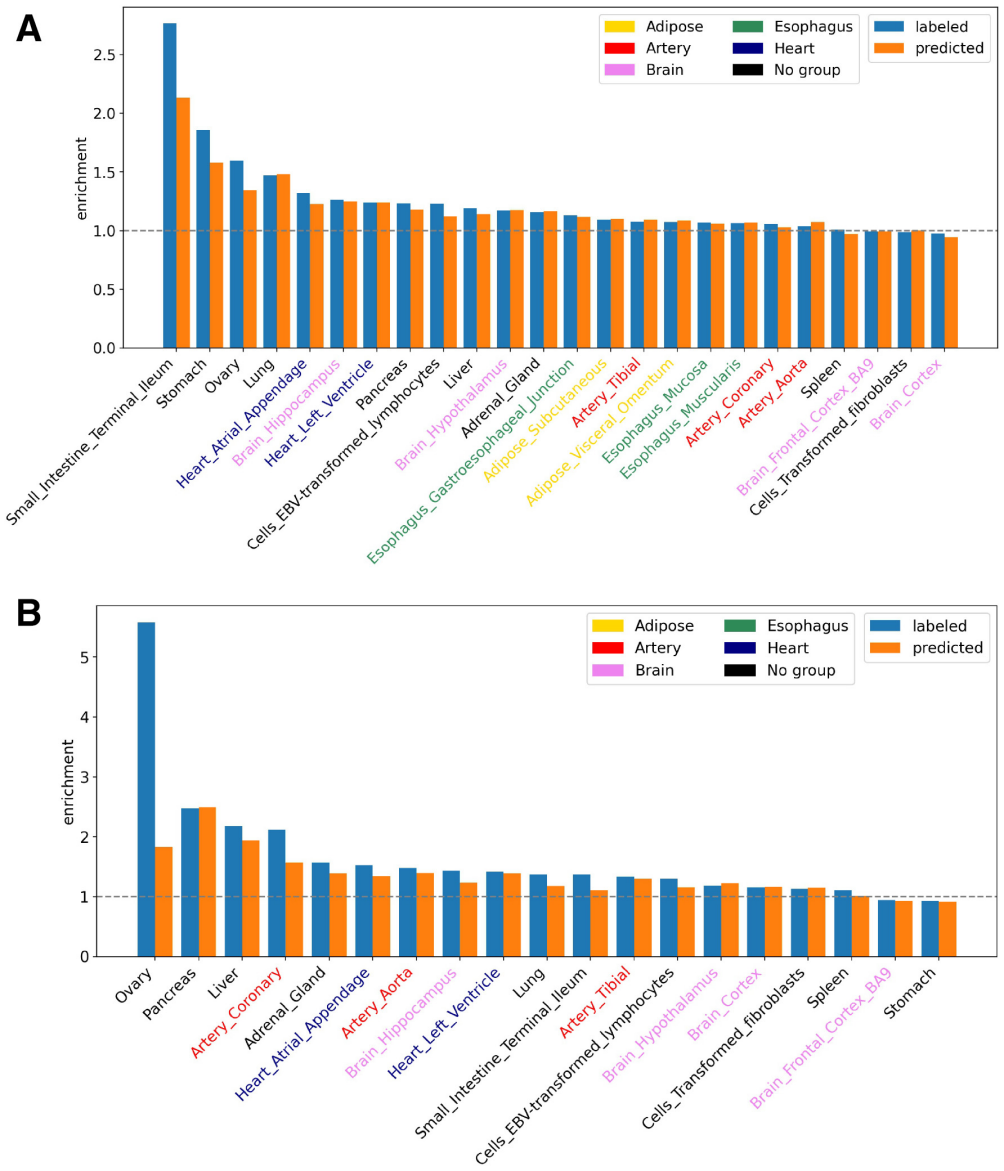
Enrichment analysis of true and predicted regulatory nc-sindels in both (**A**) PE interactions and (**B**) PP interactions among common tissues between GTEx and pcHi-C data from 3DIV

For common tissues between GTEx and pcHi-C data from 3DIV, we perform the enrichment analysis of true and predicted regulatory nc-sindels in both PE and PP (Fig. 7A and B). We find that both true and predicted labels are enriched in PE among 20 out of 24 tissues (OR >1), and are enriched in PP among 16 out of 19 tissues (OR >1). Moreover, the overall enrichment pattern between true labels and predicted labels is similar, while the overall enrichment of predicted labels is slightly lower. For PE, both true and predicted labels are significantly enriched in top-ranked tissues such as ‘Small Intestine Terminal Ileum’ (OR = 2.77 and *P*-value = $2.74\times 10^{-5}$ for true labels; OR = 2.13 and *P*-value = 0.002 for predicted labels), ‘Stomach’ (OR = 1.86 and *P*-value = $1.15\times 10^{-9}$ for true labels, OR = 1.58 and *P*-value=$7.97\times 10^{-6}$ for predicted labels) and ‘Lung’ (OR = 1.47 and *P*-value = $8.79\times 10^{-7}$ for true labels; OR = 1.48; *P*-value = $9.39\times 10^{-7}$ for predicted labels). In contrast, true labels but not predicted labels are significantly enriched in ‘Ovary’ (OR = 1.59 and *P*-value = 0.035 for true labels; OR = 1.34 and *P*-value = 0.19 for predicted labels). For PP, true labels are more enriched in ‘Ovary’ (OR = 5.58 and *P*-value = 0.002), ‘Pancreas’ (OR = 2.48 and *P*-value = $2.10\times 10^{-5}$), ‘Liver’ (OR = 2.18 and *P*-value = 0.005) and Artery Coronary (OR = 2.12 and *P*-value = $6.92\times 10^{-4}$). Notably, the enrichment of predicted labels in ‘Ovary’ is much lower (OR = 5.58 for true labels versus OR = 1.83 for predicted labels). Interestingly, we find true labels and predicted labels are enriched in ‘Ovary’ in both PE and PP.

**Fig. 8.**
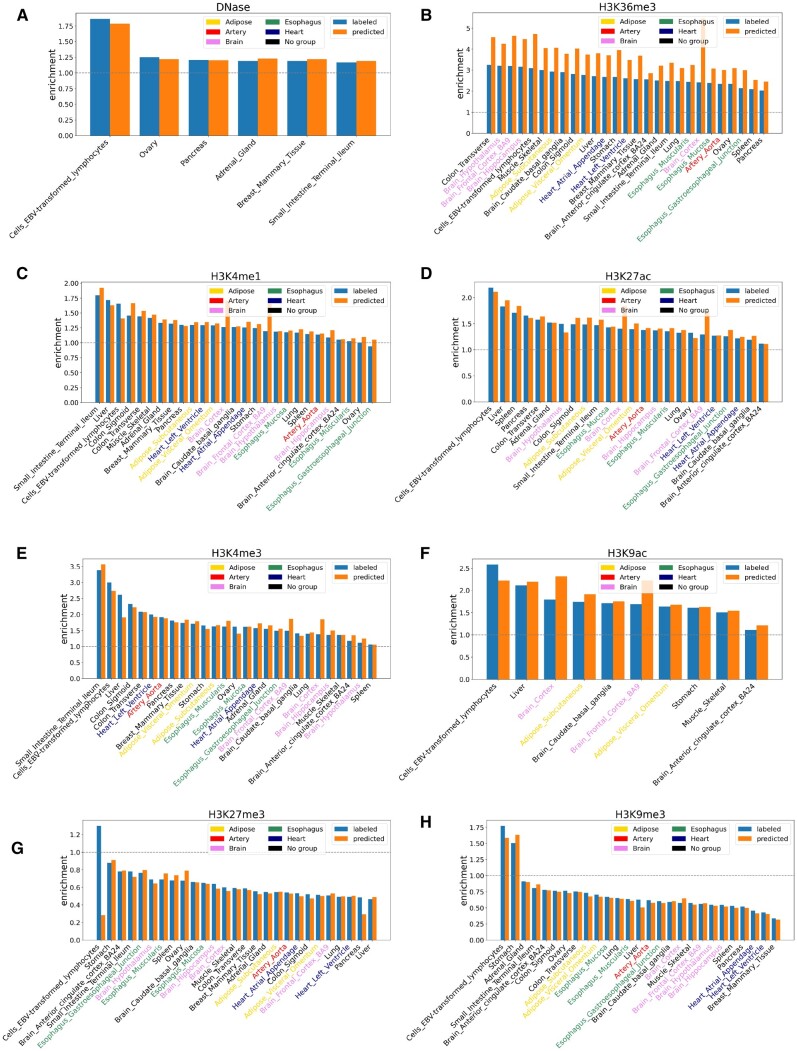
Enrichment analysis of true and predicted regulatory nc-sindels in peak regions among common tissues between GTEx and ChIP-seq data from Roadmap Epigenomics. (**A**) DNase (**B**)H3K36me3 (**C**) H3K4me1 (**D**) H3K27ac (**E**) H3K4me3 (**F**) H3K9ac (**G**) H3K27me3 (**H**) H3K9me3

Similarly, for common tissues between GTEx and ChIP-seq data from Roadmap Epigenomics, we also perform the enrichment analysis of true and predicted regulatory nc-sindels in the peak regions. The ChIP-seq data profile open chromatin, five active histone marks and two repressive histone marks (Fig. 8A–H). As a result, both true labels and predicted labels are enriched in open chromatin (OR >1) and achieve comparable enrichment across all tissues (Fig. 8A). Similarly, both true and predicted labels are significantly enriched in H3K36me3, which are associated with actively transcribed genes, across all tissues (OR >1 and *P*-value <0.05) (Fig. 8B). Importantly, predicted labels show a much higher enrichment for all tissues (median OR = 3.71 for predicted labels versus median OR = 2.62 for true labels). Though true labels are defined based on statistical significance, the significant nc-sindels are not necessarily the functional ones. Therefore, this observation indicates that TIVAN-indel may improve the prediction for the regulatory nc-sindels.

Moreover, both true and predicted labels are enriched in H3K4me1 and H3K27ac, which are histone marks associated with enhancer activation, across most tissues in a similar enrichment pattern (OR >1) (Fig. 8C and D). Interestingly, the enrichment of predicted labels is higher in ‘Brain Cortex’ and ‘Brain Frontal Cortex BA9’ in both H3K4me1 (OR = 1.71 and *P*-value = $4.75\times 10^{-11}$ for predicted labels versus OR = 1.27 and *P*-value = $3.45\times 10^{-3}$ for true labels in ‘Brain Cortex’; OR = 1.66 and *P*-value = $1.22\times 10^{-7}$ for predicted labels versus OR = 1.20 and *P*-value = $6.02\times 10^{-2}$ for true labels in ‘Brain Frontal Cortex BA9’) and H3K27ac (OR = 1.84 and *P*-value = $6.35\times 10^{-13}$ for predicted labels versus OR = 1.40 and *P*-value = $5.96\times 10^{-5}$ for true labels in ‘Brain Cortex’; OR = 1.75 and *P*-value = $3.24\times 10^{-8}$ for predicted labels versus OR = 1.29 and *P*-value = $1.11\times 10^{-2}$ for true labels in ‘Brain Frontal Cortex BA9’). Similar to active histone marks associated with enhancer activation, both true and predicted labels are enriched in H3K4me3 and H3K9ac, which are histone markers associated with promoter activation, across all tissues in a similar enrichment pattern (OR >1) (Fig. 8E and F). In addition, the enrichment of predicted labels is higher in ‘Brain Cortex’ and ‘Brain Frontal Cortex BA9’ in both H3K4me3 (OR = 1.84 and *P*-value = $4.36\times 10^{-6}$ for predicted labels versus OR = 1.38 and *P*-value = $1.73\times 10^{-2}$ for true labels in ‘Brain Cortex’; OR = 1.86 and *P*-value = $5.11\times 10^{-5}$ for predicted labels versus OR = 1.49 and *P*-value = $9.42\times 10^{-3}$ for true labels in ‘Brain Frontal Cortex BA9’) and H3K9ac (OR = 2.32 and *P*-value = $2.67\times 10^{-13}$ for predicted labels versus OR = 1.79 and *P*-value = $2.44\times 10^{-7}$ for true labels in ‘Brain Cortex’; OR = 2.22 and *P*-value = $8.39\times 10^{-10}$ for predicted labels and OR = 1.69 and *P*-value = $5.08\times 10^{-5}$ in ‘Brain Frontal Cortex BA9’). Again, the higher enrichment of predicted labels in histone marks associated with both promoter activation and enhancer activation indicates that TIVAN-indel may improve the prediction for the regulatory nc-sindels in ‘Brain Cortex’ and ‘Brain Frontal Cortex BA9’.

In contrast, both true and predicted labels are depleted in repressive chromatin marks including H3K27me3 and H3K9me3 across most tissues (OR <1) (Fig. 8G and H). For H3K9me3, both true and predicted labels are depleted in all tissues except for ‘Cells EBV-transformed lymphocytes’ and ‘Stomach’. For H3K27me3, predicted labels are depleted while true labels are enriched in ‘Cells EBV-transformed lymphocytes’ (OR = 0.28 for predicted labels versus OR = 1.30 for true labels). The depletion of predicted labels indicates that TIVAN-indel may improve the prediction for regulatory nc-sindels in ‘Cells EBV-transformed lymphocytes’.

### 3.7 Computational time and precomputed scores

We evaluate the computational time for both training TIVAN-indel on different sample sizes and testing on all nc-sindels in the 1000 Genomes Project (Auton *et al.*, 2015). The training time for TIVAN-indel costs ∼4 min and ∼380 MB memory for a small sample size (Uterus, 2258), ∼43 min and ∼2 GB for a medium sample size (Adipose Visceral Omentum, 16724) and ∼1 h 45 min and ∼4.5 GB memory for a large sample size (Adipose Subcutaneous, 39524). In the testing phase, it takes ∼4 h 30 min for calculating the functional scores for ∼35.2 million nc-sindels in 1000 Genomes Project (hg19) for one tissue. All the computational time and memory cost are evaluated on the computation platform with configurations such as CPU [Intel(R) Xeon(R)], Clock speed (2.5 GHz), Memory (256 GB) and Cores (12). To facilitate the usage of TIVAN-indel, we provide the precomputed functional scores for all nc-sindels in the 1000 Genomes Project across 44 tissues on the github webpage. With the provided R scripts on the github webpage, users can easily and quickly retrieve the functional scores for query sindels in one or multiple tissues.

## 4 Conclusion

We develop TIVAN-indel, which is a genome-wide variant annotation tool that assigns higher scores to nc-sindels that are more likely to alter the expression levels of nearby genes. In contrast to existing approaches such as CADD and FATHMM-indel, which focus on pathogenicity or deleteriousness, TIVAN-indel can interpret nc-indels’ molecular impact in gene expression and thus can offer a more comprehensive mechanistic understanding of their functional role in disease pathogenesis. Importantly, TIVAN-indel is a complementary tool to identify regulatory nc-indels considering the limitation of current sequencing experiments such as the small sample size, low MAFs and small effect size.

The advantages of TIVAN-indel lie in two aspects. First, it leverages a comprehensive set of tissue-specific nc-sindels from GTEx to train a tissue-specific model. This is important because gene expression varies in different tissues and a nc-sindel may affect the nearby gene expression in a tissue-specific manner. Second, TIVAN-indel utilizes a rich feature set, which includes both generic functional annotations derived from CADD and large-scale epigenomic profiles predicted by a deep learning model (i.e. DanQ). Especially, the epigenomic profiles, which are predicted from both reference and alternative genomic sequences, provide the cell type-specific characterization of possible mechanistic insights into how a nc-sindel impacts the gene expression, which in turn improve the prediction for regulatory nc-sindels. Using the GTEx data, we demonstrate that using both feature sets improve the prediction performance compared with using either feature set.

We apply both within-tissue and cross-tissue approaches to evaluate TIVAN-indel and its competing methods on the GTEx data. For within-tissue approach, we adopt 5-fold cross-validation to evaluate the prediction performance in the same tissue. For cross-tissue approach, we train the model using one tissue and test the model in the other tissues. To allow a fair comparison, we reimplement CADD and FATHMM-indel using the GTEx data to allow the same training and testing sets between TIVAN-indel and the competing methods. Still, TIVAN-indel significantly outperforms these methods in both ways of evaluation. To further demonstrate the robustness of TIVAN-indel, we train TIVAN-indel along with its competing methods using the ‘Whole Blood’ tissue from the GTEx data and evaluate all methods using 15 immune cell types from an independent study named DICE. Similarly, TIVAN-indel achieves the best prediction performance across all immune cell types. This independent evaluation further confirms the superiority of TIVAN-indel in predicting regulatory nc-indels.

Moreover, we perform an enrichment analysis for nc-sindels by hypothesizing that regulatory nc-sindels need to be enriched in key regulatory regions such as enhancer, promoter and chromatin interaction in order to have an impact on gene expression. As expected, both true and predicted labels are enriched in PP and PE interactions across most tissues. Similarly, both true and predicted labels are enriched in histone marks associated with promoter, enhancer and gene activation. In contrast, they are depleted in repressive histone marks associated with downregulation of gene expression. Importantly, both true and predicted labels share a similar enrichment pattern in open chromatin, active histone marks and repressive histone marks, which indicates a good prediction made by TIVAN-indel. Interestingly, the enrichment of predicted labels is higher in active histone marks associated gene activation across all tissues, and active histone marks associated with promoter and enhancer activation in ‘Brain Cortex-’ and ‘Brain Frontal Cortex BA9’. Similarly, predicted labels are depleted while true labels are enriched in ‘Cells EBV-transformed lymphocytes’ in H3K27me3. These observations indicate that TIVAN-indel can accurately predict regulatory nc-sindels, which are otherwise missed by the statistical measurement.

We deliver TIVAN-indel as an open-source Python toolkit, which allows users to train the model and predict the functional scores for given sc-indels. To make TIVAN-indel widely accessible to the research community, we provide the precomputed functional scores for ∼35.2 million nc-sindels in the 1000 Genomes Project across 44 tissues. The functional scores can be potentially used as the functional weights in the rare variant association analysis of nc-sindels in whole-genome sequencing studies such as the STAAR framework (Gaynor *et al.*, 2022; Li *et al.*, 2020, 2022b). The functional scores can be used to prioritize regulatory nc-sindels, which can help narrow down the candidates for experimental validation by using Massively Parallel Reporter Assay or CRISPR-Cas9. This application makes TIVAN-indel practically useful considering current high-throughput sequencing technologies are still infeasible to validate all nc-sindels in human genome due to technical and financial challenges.

In this work, TIVAN-indel focus on predicting regulatory nc-sindels that may affect nearby gene expression by leveraging the GTEx summary statistics data, which are derived from the cis-eQTL analysis working on matched gene expression data and genotype data across multiple tissues. Given the readily availability of matched genotype data and other types of functional omics data, TIVAN-indel can be easily extended to predict nc-sindels that impact on molecular phenotypes such as chromatin accessibility, DNA methylation and transcription factor binding. Therefore, multiple sets of functional scores can be developed to measure the functional potential of nc-sindels in different aspects of molecular functions, which can provide a comprehensive functional landscape for nc-sindels in human genome.

The nature of supervised learning makes TIVAN-indel dependent on the quality of functional nc-sindels identified from the cis-QTL analysis for constructing the training sets. In the future, we plan to develop an unsupervised version of TIVAN-indel, which does not rely on the labeled nc-sindels and can integrate and collapse multiple correlated functional annotations into one functional score. A further interest also includes integrating both supervised and unsupervised versions of TIVAN-indel to improve prediction accuracy.

## Supplementary Material

btad060_Supplementary_Data
