# Supplementary material for: TIVAN-indel: a computational framework for annotating and predicting non-coding regulatory small insertions and deletions
Source: Bioinformatics. 2023 Jan 27;39(2):btad060. doi: 10.1093/bioinformatics/btad060 (PMC9900211; doi:10.1093/bioinformatics/btad060)
Supplement: btad060_Supplementary_Data [file btad060_supplementary_data.pdf]

Supplementary materials for TIVAN-indel: A computational framework for annotating and predicting noncoding regulatory small insertions and deletions

1 Supplementary tables

Table S1: Summary of the numbers of labeled nc-sindels in 44 tissues in GTEx

Tissue	#Samples
Adipose Subcutaneous	39524
Adipose Visceral Omentum	16724
Adrenal Gland	11052
Artery Aorta	26777
Artery Coronary	7649
Artery Tibial	39095
Brain Anterior cingulate cortex BA24	3226
Brain Caudate basal ganglia	7231
Brain Cerebellar Hemisphere	10148
Brain Cerebellum	13809
Brain Cortex	7540
Brain Frontal Cortex BA9	5728
Brain Hippocampus	3021
Brain Hypothalamus	3135
Brain Nucleus accumbens basal ganglia	5863
Brain Putamen basal ganglia	4358
Breast Mammary Tissue	15731
Cells EBV-transformed lymphocytes	10236
Cells Transformed fibroblasts	43632
Colon Sigmoid	9972
Colon Transverse	16671
Esophagus Gastroesophageal Junction	10225
Esophagus Mucosa	33910
Esophagus Muscularis	31487
Heart Atrial Appendage	15059
Heart Left Ventricle	18523
Liver	4978
Lung	31046
Muscle Skeletal	36293
Nerve Tibial	45474
Ovary	4349
Pancreas	16630
Pituitary	6337
Prostate	4172
Skin Not Sun Exposed Suprapubic	21141
Skin Sun Exposed Lower leg	39416
Small Intestine Terminal Ileum	3289
Spleen	7860
Stomach	13390
Testis	30251
Thyroid	45561
Uterus	2258
Vagina	2390
Whole Blood	33580

Table S2: Summary of CADD functional annotations used for TIVAN-indel

	Annotation	Annotation Class	Annotation Type
1	Consequence	VEP consequence, priority selected by potential impact	categorical
2	ConsScore	Custom deleterious score assigned to Consequence	numeric
3	GC	Percent GC in a window of $+/-$ 75bp	numeric
4	CpG	Percent CpG in a window of $+/-$ 75bp	numeric
5	minDistTSS	Distance to closest Transcribed Sequence Start (TSS)	numeric
7	minDistTSE	Distance to closest Transcribed Sequence Start (TSE)	numeric
7	priPhCons	Primate PhastCons conservation score	numeric
8	mamPhCons	Mammalian PhastCons conservation score	numeric
9	verPhCons	Vertebrate PhastCons conservation score	numeric
10	priPhyloP	Primate PhyloP score	numeric
11	mamPhyloP	Mammalian PhyloP score	numeric
12	verPhyloP	Vertebrate PhyloP score	numeric
13	bStatistic	Background selection score	numeric
14	cHmmTssA	Number of 48 cell types in chromHMM state	numeric
15	cHmmTssAFlnk	Number of 48 cell types in chromHMM state	numeric
16	cHmmTxFlnk	Number of 48 cell types in chromHMM state	numeric
17	cHmmTx	Number of 48 cell types in chromHMM state	numeric
18	cHmmTxWk	Number of 48 cell types in chromHMM state	numeric
19	cHmmEnhG	Number of 48 cell types in chromHMM state	numeric
20	cHmmEnh	Number of 48 cell types in chromHMM state	numeric
21	cHmmZnfRpts	Number of 48 cell types in chromHMM state	numeric
22	cHmmHet	Number of 48 cell types in chromHMM state	numeric
23	cHmmTssBiv	Number of 48 cell types in chromHMM state	numeric
24	cHmmBivFlnk	Number of 48 cell types in chromHMM state	numeric
25	cHmmEnhBiv	Number of 48 cell types in chromHMM state	numeric
26	cHmmReprPC	Number of 48 cell types in chromHMM state	numeric
27	cHmmReprPCWk	Number of 48 cell types in chromHMM state	numeric
28	cHmmQuies	Number of 48 cell types in chromHMM state	numeric
29	GerpN	Neutral evolution score defined by GERP++	numeric
30	GerpS	Rejected Substitution score defined by GERP++	numeric
31	EncH3K27Ac	Encode H3K27ac levels (from 14 cell lines)	numeric
32	EncH3K4Me1	Encode H3K4me1 levels (from 14 cell lines)	numeric
32	EncH3K4Me3	Encode H3K4me3 levels (from 14 cell lines)	numeric
34	EncNucleo	Encode nucleosome occupancy levels (from 14 cell lines)	numeric
35	Segway	transcriptome/epigenome (ENCODE/Roadmap)	categorical
36	Dist2Mutation	Distance between the closest BRAVO SNV up and downstream	numeric
37	Freq100bp	Number of frequent (MAF $>$0.05) BRAVO SNV in 100 bp window nearby	numeric
38	Rare100bp	Number of rare (MAF $<$ 0.05) BRAVO SNV in 100 bp window nearby	numeric
39	Sngl100bp	Number of single occurrence BRAVO SNV in 100 bp window nearby	numeric
40	Freq1000bp	Number of frequent (MAF $>$0.05) BRAVO SNV in 1000 bp window nearby	numeric
41	Rare1000bp	Number of rare (MAF $<$ 0.05) BRAVO SNV in 1000 bp window nearby	numeric
42	Sngl1000bp	Number of single occurrence BRAVO SNV in 1000 bp window nearby	numeric
43	Freq10000bp	Number of frequent (MAF $>$0.05) BRAVO SNV in 10000 bp window nearby	numeric
44	Rare10000bp	Number of rare (MAF $<$ 0.05) BRAVO SNV in 10000 bp window nearby	numeric
45	Sngl10000bp	Number of single occurrence BRAVO SNV in 10000 bp window nearby	numeric

Table S3: Summary of chromatin interactions (PP and PE) in the common tissues between GTEx and pcHi-C

Tissue	#Interactions	tissue	#PE (FDR < 0.1)	#PP (FDR < 0.1)
Adipose Subcutaneous	41363	Fat	56057	×
Adipose Visceral Omentum	17567	Fat	56057	×
Adrenal Gland	11626	Adrenal Gland	2556	5421
Artery Aorta	28031	Aorta	5224	788
Artery Coronary	8050	Aorta	5224	788
Artery Tibial	40984	Aorta	5224	788
Brain Anterior cingulate cortex BA24	3425	Dorsorateral prefrontal cortex	26084	6224
Brain Caudate basal ganglia	7643	Dorsorateral prefrontal cortex	26084	6224
Brain Cerebellar Hemisphere	10685	Dorsorateral prefrontal cortex	26084	6224
Brain Cerebellum	14543	Dorsorateral prefrontal cortex	26084	6224
Brain Cortex	7973	Dorsorateral prefrontal cortex	26084	6224
Brain Frontal Cortex BA9	6054	Dorsorateral prefrontal cortex	26084	6224
Brain Hippocampus	3201	Hippocampus	17434	2519
Brain Hypothalamus	3324	Hippocampus	17434	2519
Brain Nucleus accumbens basal ganglia	6183	Dorsorateral prefrontal cortex	26084	6224
Brain Putamen basal ganglia	4617	Dorsorateral prefrontal cortex	26084	6224
Breast Mammary Tissue	16546	×	-	-
Cells EBV-transformed lymphocytes	10756	GM12878+GM19240 Lymphoblastoid Cell Line	4903	4446
Cells Transformed fibroblasts	45580	Fibroblast cells	13217	4156
Colon Sigmoid	10518	Sigmoid colon	×	10338
Colon Transverse	17565	Sigmoid colon	×	10338
Esophagus Gastroesophageal Junction	10758	Esophagus	20049	×
Esophagus Mucosa	35559	Esophagus	20049	×
Esophagus Muscularis	33027	Esophagus	20049	×
Heart Atrial Appendage	15849	Left Ventricle	1565	968
Heart Left Ventricle	19472	Left Ventricle	1565	968
Liver	5252	Liver	5325	706
Lung	32528	Lung	1188	661
Muscle Skeletal	37990	×	-	-
Nerve Tibial	47555	×	-	-
Ovary	4586	Ovary	1105	187
Pancreas	17451	Pancreas	2594	396
Pituitary	6703	×	-	-
Prostate	4410	×	-	-
Skin Not Sun Exposed Suprapubic	22215	×	-	-
Skin Sun Exposed Lower leg	41294	×	-	-
Small Intestine Terminal Ileum	3500	Small Bowel	972	704
Spleen	8307	Spleen	22392	5866
Stomach	14100	Gastric tissue	1610	900
Testis	31732	×	-	-
Thyroid	47647	×	-	-
Uterus	2398	×	-	-
Vagina	2554	×	-	-
Whole Blood	35143	×	-	-

Table S4: Summary of ChIP-seq data in the common tissues between GTEx and Roadmap Epigenomics

Tissue (GTEx)	#Peaks	Tissue (Roadmap)	DNase	H3K27me3	H3K4me1	H3K9ac	H3K27ac	H3K36me3	H3K4me3	H3K9me3
Adipose Subcutaneous	41363	Adipose Nuclei	×	187394	257141	98296	120547	252686	83253	208451
Adipose Visceral Omentum	17567	Adipose Nuclei	×	187394	257141	98296	120547	252686	83253	208451
Adrenal Gland	11626	Fetal Adrenal Gland	381299	287466	276138	×	155447	292480	33369	211821
Artery Aorta	28031	Aorta	×	47224	132931	×	130935	62301	37104	71996
Artery Coronary	8050	×	-	-	-	-	-	-	-	-
Artery Tibial	40984	×	-	-	-	-	-	-	-	-
Brain Anterior cingulate cortex BA24	3425	Brain Cingulate Gyrus	×	71546	255504	125356	166221	198074	74743	89353
Brain Caudate basal ganglia	7643	Brain Anterior Caudate	×	37011	271201	166608	209550	224169	85705	149449
Brain Cerebellar Hemisphere	10685	×	-	-	-	-	-	-	-	-
Brain Cerebellum	14543	×	-	-	-	-	-	-	-	-
Brain Cortex	7973	Brain Dorsolateral Prefrontal Cortex	×	108957	218893	102626	198908	187780	71215	90299
Brain Frontal Cortex BA9	6054	Brain Dorsolateral Prefrontal Cortex	×	108957	218893	102626	198908	187780	71215	90299
Brain Hippocampus	3201	Brain Hippocampus Middle	×	118969	224117	×	150278	189289	75240	124885
Brain Hypothalamus	3324	Brain Hippocampus Middle	×	118969	224117	×	150278	189289	75240	124885
Brain Nucleus accumbens basal ganglia	6183	×	-	-	-	-	-	-	-	-
Brain Putamen basal ganglia	4617	×	-	-	-	-	-	-	-	-
Breast Mammary Tissue	16546	Breast vHMEC	239398	121677	344219	×	×	212637	51855	188686
Cells EBV-transformed lymphocytes	10756	Lymphoblastoid Cells	224379	401	115050	47553	75909	127502	67368	27438
Cells Transformed fibroblasts	45580	×	-	-	-	-	-	-	-	-
Colon Sigmoid	10518	Sigmoid Colon	×	135997	110437	×	179713	279430	59431	111694
Colon Transverse	17565	Sigmoid Colon	×	135997	110437	×	179713	279430	59431	111694
Esophagus Gastroesophageal Junction	10758	Esophagus	×	50452	237103	×	149589	239923	50117	229209
Esophagus Mucosa	35559	Esophagus	×	50452	237103	×	149589	239923	50117	229209
Esophagus Muscularis	33027	Esophagus	×	50452	237103	×	149589	239923	50117	229209
Heart Atrial Appendage	15849	Left Ventricle	×	108042	254975	×	150090	271995	39466	156533
Heart Left Ventricle	19472	Left Ventricle	×	108042	254975	×	150090	271995	39466	156533
Liver	5252	Liver	×	86572	229385	102736	115620	296963	85967	249912
Lung	32528	Lung	×	49060	295061	×	213427	322694	76220	401423
Muscle Skeletal	37990	Skeletal Muscle Male	×	180943	234626	137343	×	181211	76955	65435
Nerve Tibial	47555	×	-	-	-	-	-	-	-	-
Ovary	4586	Ovary	346550	69500	276899	×	155632	315170	39533	99286
Pancreas	17451	Pancreas	252470	2004	281414	×	81234	256002	51479	218849
Pituitary	6703	×	-	-	-	-	-	-	-	-
Prostate	4410	×	-	-	-	-	-	-	-	-
Skin Not Sun Exposed Suprapubic	22215	×	-	-	-	-	-	-	-	-
Skin Sun Exposed Lower leg	41294	×	-	-	-	-	-	-	-	-
Small Intestine Terminal Ileum	3500	Small Intestine	270628	170557	171197	×	189419	139581	45684	53091
Spleen	8307	Spleen	×	13501	315506	×	124962	267783	153962	213867
Stomach	14100	Stomach Mucosa	×	111365	212099	84198	×	67217	46348	17149
Testis	31732	×	-	-	-	-	-	-	-	-
Thyroid	47647	×	-	-	-	-	-	-	-	-
Uterus	2398	×	-	-	-	-	-	-	-	-
Vagina	2554	×	-	-	-	-	-	-	-	-
Whole Blood	35143	×	-	-	-	-	-	-	-	-

Table S5: Summary of the numbers of labeled nc-sindels in 15 immune cell types in DICE

Tissue	#Sample Size
Naive B cells	12312
Naive CD4+ T cells	14342
Stimulated CD4+ T cells	9419
Naive CD8+ T cells	14976
Stimulated CD8+ T cells	9845
Non-classic monocytes (M2)	11791
Classic monocytes (monocytes)	12835
NK cells	9302
Tfh	14019
Th1 cells	10451
Th17 cells	15317
Th2 cells	14470
Th1/17 cells (THSTAR)	13296
Memory Treg cells	13750
Naive Treg cells	15496

2 Supplementary figures

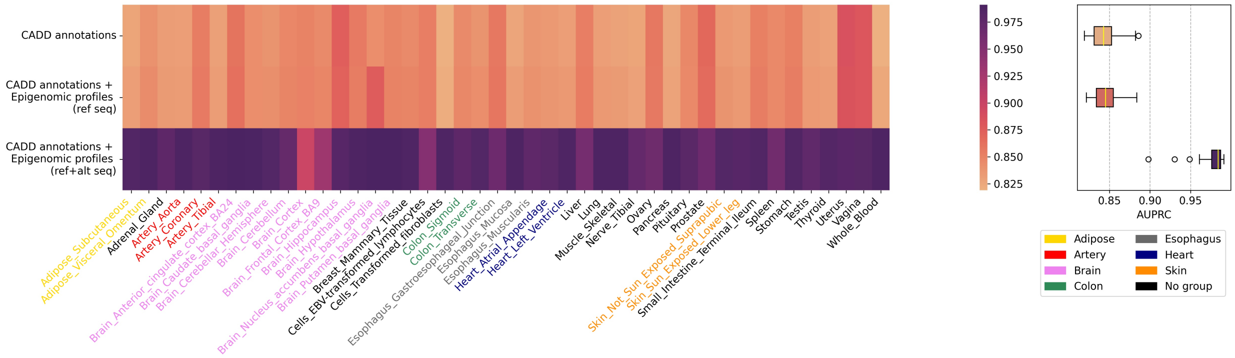

Figure S1: Comparison of three feature sets of TIVAN-indel: (i) 45 CADD annotations; (ii) 45 CADD annotations+919 epigenomic profiles predicted from the reference genomic sequence; (iii) 45 CADD annotations+919×2 epigenomic profiles predicted from both the reference and alternative genomic sequence. We evaluate the prediction performance using five-fold cross-validation across 44 tissues in GTEx. The median of AUPRC across 44 tissues is reported for each feature set.

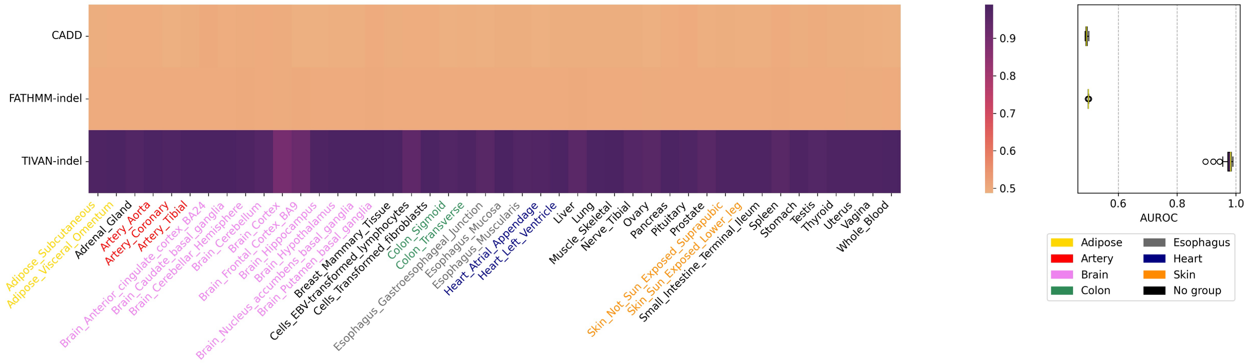

Figure S2: Comparison between TIVAN-indel, CADD and FATHMM-indel for 44 tissues in GTEx using the precomputed scores from CADD and FATHMM-indel. AUROC is reported for each tissue.

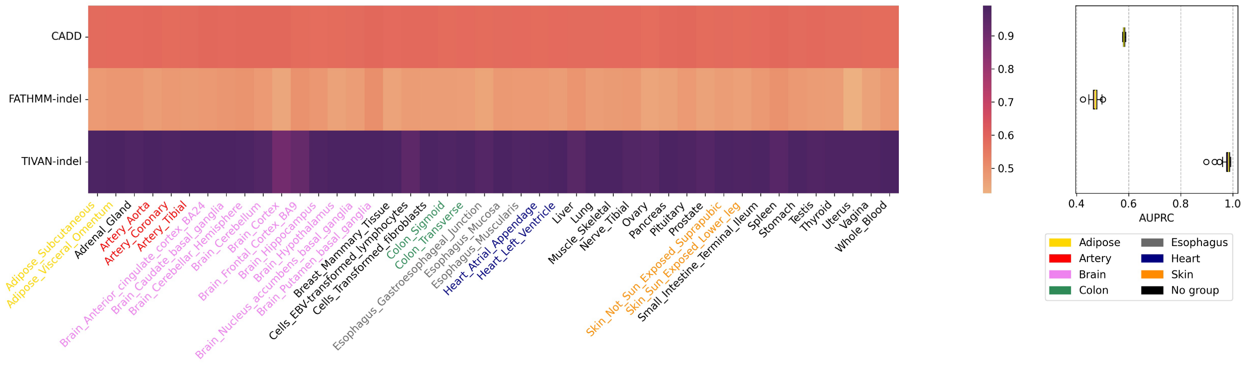

Figure S3: Comparison between TIVAN-indel, CADD and FATHMM-indel for 44 tissues in GTEx using the precomputed scores from CADD and FATHMM-indel. AUPRC is reported for each tissue.

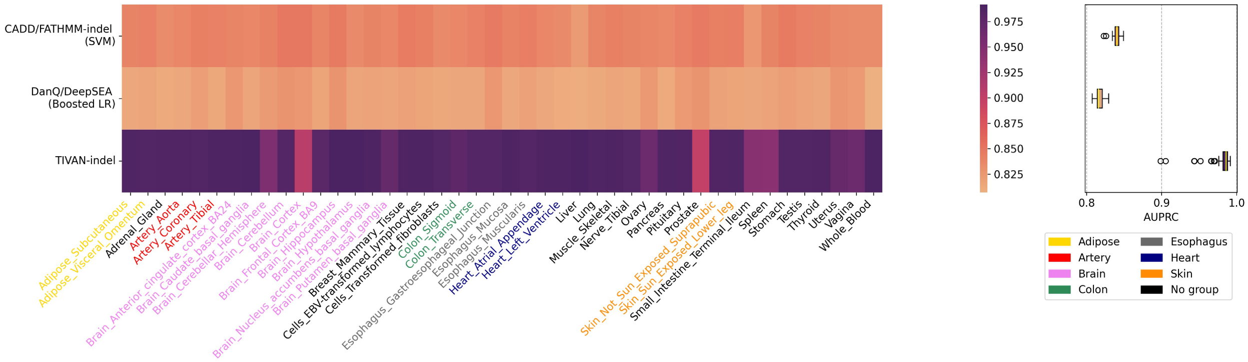

Figure S4: Comparison between TIVAN-indel, CADD/FATHMM-indel (SVM) and DanQ/DeepSEA (Boosted LR) using the within-tissue approach for 44 tissues in GTEx. Each method is trained and tested using five-fold cross-validation for each tissue. AUPRC is reported for each tissue.

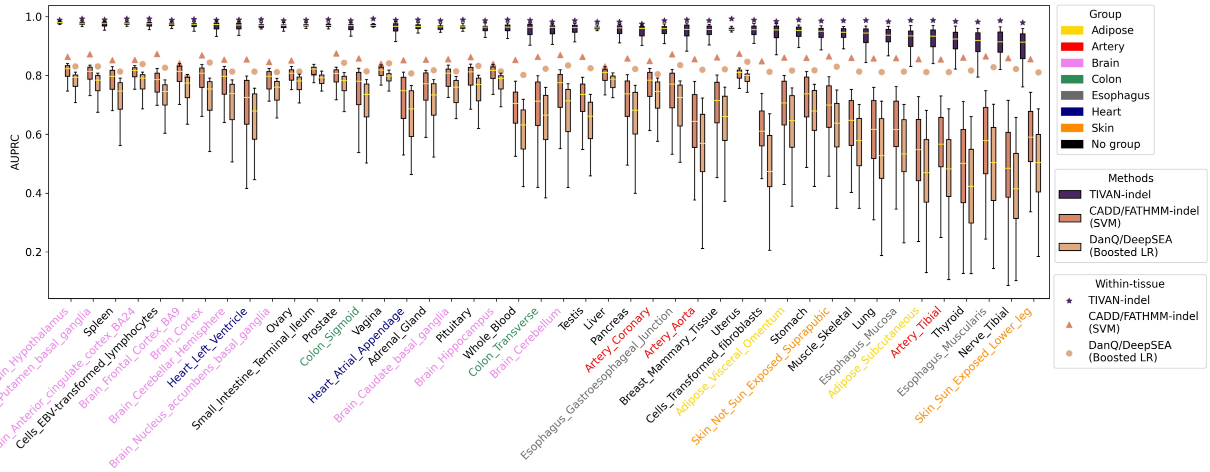

Figure S5: Comparison between TIVAN-indel, CADD/FATHMM-indel (SVM) and DanQ/DeepSEA (Boosted LR) using the cross-tissue approach for 44 tissues in GTEx, where each method is trained using one tissue and tested on the other 43 tissues. The overlapped nc-sindels between the training and testing sets are removed from the testing set. For each tissue, AUPRC is reported for the other 43 tissues as demonstrated in the boxplot. The asterisk denotes the AUPRC calculated from the within-tissue approach. 44 tissues are colored in 8 tissue groups.

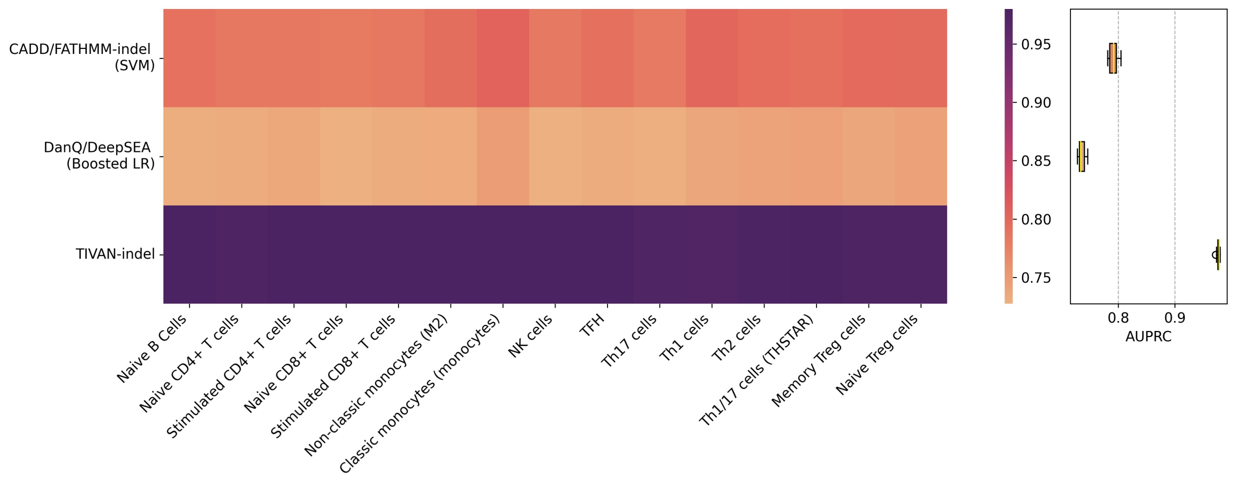

Figure S6: Comparison between TIVAN-indel, CADD/FATHMM-indel (SVM) and DanQ/DeepSEA (Boosted LR) by training the model on the "Whole blood" in GTEx and testing the model on 15 immune cell types in DICE. The overlapped nc-sindels between training and testing sets are removed from the testing set. The AUPRC is reported for each cell type.

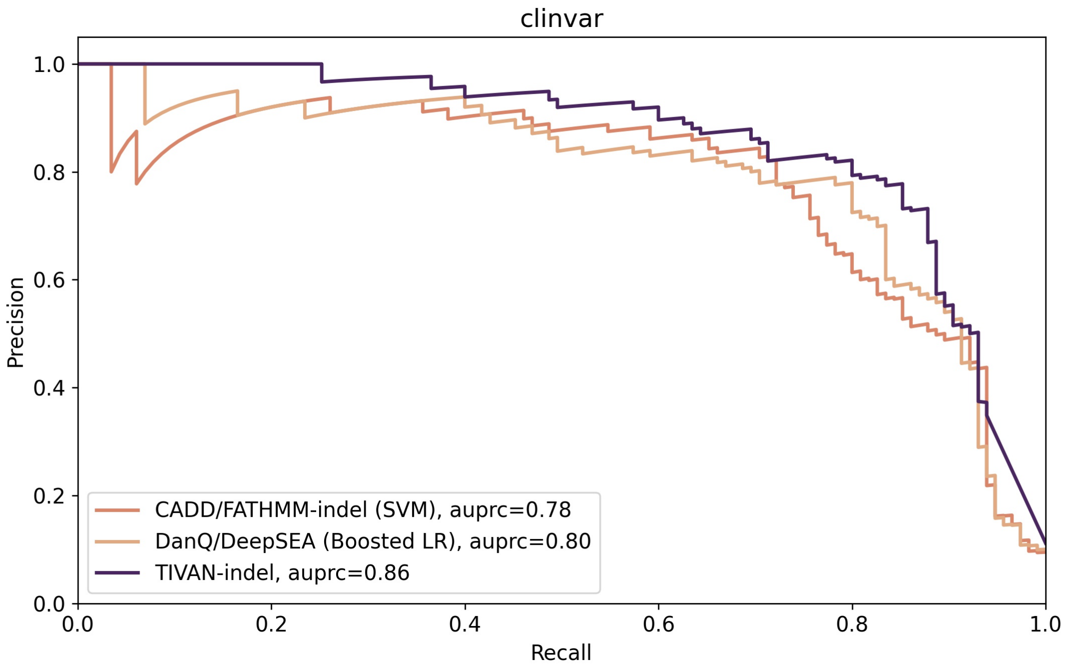

Figure S7: Comparison between TIVAN-indel, CADD/FATHMM-indel (SVM) and DanQ/DeepSEA (Boosted LR) on predicting pathogenic nc-sindels in ClinVar. The AUPRC is reported.
